# Lipid Homeostasis and Its Links With Protein Misfolding Diseases

**DOI:** 10.3389/fnmol.2022.829291

**Published:** 2022-03-25

**Authors:** Michele Vendruscolo

**Affiliations:** Centre for Misfolding Diseases, Department of Chemistry, University of Cambridge, Cambridge, United Kingdom

**Keywords:** lipid homeostasis, protein homeostasis, protein condensation, Alzheimer’s disease, Parkinson’s disease

## Abstract

The maintenance of lipid homeostasis is essential for the normal functioning of living organisms. Alterations of the lipid homeostasis system remodel the composition of the lipidome, potentially leading to the formation of toxic lipid species. In turn, lipidome changes can affect the protein homeostasis system by causing perturbations that elicit protein condensation phenomena such as protein liquid-liquid phase separation and protein aggregation. Lipids can also be more directly involved the formation of aberrant condensed states of proteins by facilitating the early events that initiate these processes and by stabilizing the condensed states themselves. These observations suggest that lipid-induced toxicity can contribute to protein misfolding diseases, including Alzheimer’s and Parkinson’s diseases. According to this view, an impairment of the lipid homeostasis system generates toxic states of lipids that disturb the protein homeostasis system and promote the formation of toxic states of proteins.

## Introduction

Aging and unbalanced lifestyles are major risk factors for a wide range of human disorders, including neurodegenerative diseases and type 2 diabetes, which are among the leading causes of death worldwide (Patterson, 2018). After decades of intense research, we have made progress in understanding the molecular origins of these disorders, which are characteristically associated with the aberrant condensation of proteins, a phenomenon that can take place via misfolding and aggregation (Knowles et al., 2014; Selkoe and Hardy, 2016) or via liquid-liquid phase separation (Hyman et al., 2014; Banani et al., 2017; Boeynaems et al., 2018; Fuxreiter and Vendruscolo, 2021). Since, however, we are still largely unable to prevent, delay or cure these disorders (Cummings et al., 2021), there is an urgent unmet need to identify and characterize in more detail the factors that contribute to the pathological condensation of proteins.

At the thermodynamic level, proteins tend to undergo condensation because at the concentrations at which they are expressed in the cells they are often close to their supersaturation limits and thus their native states are often metastable (Vecchi et al., 2020), with their conversion into dysfunctional assemblies being limited by kinetic barriers (Knowles et al., 2014; Box 1). To maintain a cellular steady state in which the majority of proteins are optimally functioning, a robust quality control system, known as protein homeostasis, has evolved to regulate the synthesis, trafficking, localizations, concentrations, interactions, degradation and conformations of these molecules (Balch et al., 2008; Brehme et al., 2014; Knowles et al., 2014; Hipp et al., 2019).

In this article, we discuss how the protein homeostasis system does work in isolation, but in coordination with the corresponding systems that regulate the behavior of the other cellular components, including lipids, nucleic acids, carbohydrates and metabolites. Characterizing these complex relationships within the overall cellular homeostasis system provides insights into the fundamental conditions and causes that promote the dysregulated condensation of proteins.

In this search, an increasing body of evidence is implicating lipids as key factors affecting protein phase behavior (Di Paolo and Kim, 2011; Galvagnion et al., 2015; Killinger et al., 2019; Fanning et al., 2020a). Multiple examples are emerging of mechanisms by which the protein and the lipid homeostasis systems affect each other, revealing how the consequences of their impairment contribute in a cooperative manner to the onset and progression of protein misfolding diseases (Di Paolo and Kim, 2011; Killinger et al., 2019; Bartels et al., 2020; Fanning et al., 2020a). As we are obtaining new information with increasing pace about the links between the protein and lipid homeostasis systems, this review identifies our current knowledge about their interconnections.

**Box 1. Liquid-liquid phase separation: Protein condensates and lipid condensates.**

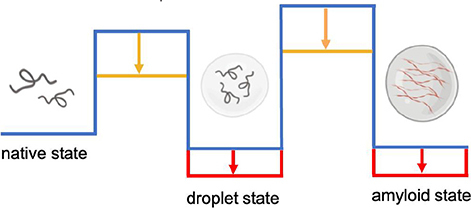

It has been recently realized that in addition to the native and amyloid states, proteins can populate a droplet state through a liquid-liquid phase separation process (Hyman et al., 2014; Banani et al., 2017; Boeynaems et al., 2018; Fuxreiter and Vendruscolo, 2021). The droplet state is associated with the formation of membraneless organelles, which play a wide range of functions in the cell (Hyman et al., 2014; Banani et al., 2017; Boeynaems et al., 2018). At the structural level, the droplet state is characterized by the condensation of proteins in a liquid-like phase, which can then evolve further into a solid-like amyloid phase. Lipids can serve both as scaffolds, by increasing the stability (red arrows) and kinetic accessibility (orange arrows) of the condensed states, and as clients, by being incorporated passively as a result of physiological or aberrant interactions during the phase separation processes. Membrane-associated proteins are particularly prone to this type of behavior. For example, synapsin has been reported to cluster synaptic vesicles by forming a condensate (Milovanovic et al., 2018), and α-synuclein to form protein droplets in the presence of lipid vesicles (Ray et al., 2020; Hardenberg et al., 2021), potentially leading to the formation of Lewy bodies (Hardenberg et al., 2021). Similarly, the E2 isoform of apolipoprotein E (ApoE2) has been shown to form condensates within the retinal pigment epithelium as possible precursors to drusen, leading to photoreceptor degeneration and vision loss in age-related macular degeneration (La Cunza et al., 2020). Liquid-liquid phase separation is a general phenomenon of central importance also for lipid systems. Lipid rafts can be thought of as membraneless lipid condensates, as they are the result of the phase separation of lipids in membranes in an ordered state rich in sphingolipids and cholesterol, which can pack quite closely through favorable hydrophobic and hydrogen bonding interactions, and a cholesterol-poor disordered state (Sezgin et al., 2017). Lipid liquid-liquid phase separation has also been observed in the membranes of the endoplasmic reticulum upon upregulation of the metabolism of saturated fatty acids, which are amenable to high-density packing (Shen et al., 2017). Furthermore, lipid droplets may originate from the nucleation of neutral lipids within the phospholipid bilayers of the membranes of the endoplasmic reticulum through liquid-liquid phase separation (Zoni et al., 2020), although upon maturation and release into the cytoplasm they acquire a lipid membrane. Similar to protein condensates, which can evolve into solid-like aggregates, also lipid condensates can acquire solid-like properties. Although lipids are soluble in the non-polar environment of the lipid membrane, they may, at least in principle, become supersaturated even within this environment and form aberrant assemblies. The formation of cholesterol plaques on the artery walls in atherosclerosis may be related to this phenomenon.

We discuss the relationships between proteinopathies, which are diseases caused by protein-induced toxicity (proteotoxicity) (Walker and LeVine, 2000), and lipidopathies, which are conditions induced by lipid-induced toxicity (lipotoxicity) (Fanning et al., 2020a), and present evidence linking lipotoxicity to proteinopathies. Through the examples of Alzheimer’s and Parkinson’s diseases, we indicate ways in which progress in understanding the interplay between the lipid and the protein homeostasis systems leads to a more detailed understanding of the mechanisms that give rise to protein misfolding diseases. In turn, this understanding reveals promising research directions to develop novel diagnostic and therapeutic interventions for these disorders.

## Lipid Homeostasis and Its Links With Protein Homeostasis

**FIGURE 1:**
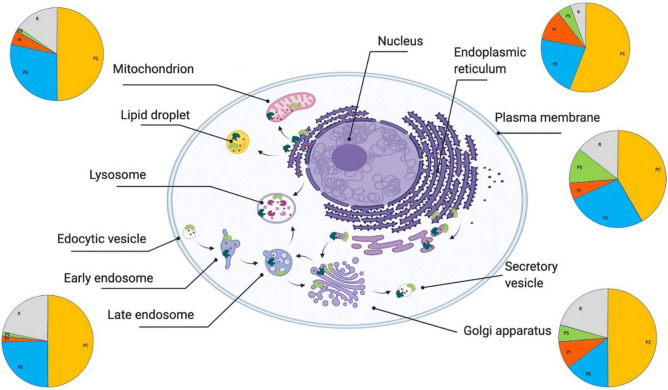
Lipid homeostasis and its links with protein homeostasis. The lipid homeostasis system is the ensemble of cellular processes (indicated by black arrows) that regulate the behavior of lipids (indicated by small red circles) in terms of their synthesis, trafficking, localizations, concentrations, interactions, and degradation (Unger et al., 2010). The lipid membranes of different organelles have different compositions (as indicated schematically by the pie charts). The major structural lipids in mammalian cell membranes are glycerophospholipids (PC, phosphatidylcholine (orange); PE, phosphatidylethanolamine (blue); PI, phosphatidylinositol (red); PS, phosphatidylserine (green), and R:rest (gray)), sphingolipids (sphingomyelin and glycosylceramide), and sterols. These lipid compositions are tightly regulated by a range of lipid-processing proteins, including enzymes (light green) and molecular chaperones (dark green), that perform lipid synthesis, which happens predominantly in the endoplasmic reticulum, Golgi and mitochondria, lipid trafficking, which includes both translocation between the two leaflets of cell membranes (flippases and scramblases) and transport through the secretory pathway, and lipid degradation.

Lipidomic studies have shown that thousands of different lipid species exist in the human lipidome (Van Meer et al., 2008), and it has been estimated that perhaps 5% of all human genes are involved in lipid synthesis (Van Meer et al., 2008). The different classes of lipids, including energy storage lipids, structural lipids, and signaling lipids, are closely interlinked through the lipid homeostasis system (Figure 1). The current mapping of the lipid homeostasis system is still incomplete, although a substantial amount of knowledge has been generated (Unger et al., 2010). In turn, lipid homeostasis is linked in a bidirectional manner with protein homeostasis. As we will discuss, the activation of specific branches of the protein homeostasis system is controlled by the state of the lipidome.

The majority of lipids in the human lipidome are made from three types of hydrocarbon-based building blocks, including fatty acids, which are carboxylic acids with aliphatic chains of various lengths, sphingosine, which is an amino alcohol with an unsaturated aliphatic chain, and sterols, which are polycyclic compounds with a core made of three cyclohexane rings and one cyclopentane ring. The main energy storage lipids are triacylglycerols, which consist of a glycerol backbone esterified with three fatty acids, and steryl esters, which consist of sterols esterified with a fatty acid. Structural lipids include glycerophospholipids, which are made by a glycerol backbone esterified with two fatty acids, sphingolipids, which typically consist of a ceramide core made by a sphingosine backbone linked to a fatty acid with an amide bond, and cholesterol. Signaling lipids include free fatty acids, including eicosanoids and docosanoids, diacylglycerols, which consist of a glycerol backbone esterified with two fatty acids, phosphoinositides, which are phosphorylated forms of phosphatidylinositol (an inositol-containing phospholipid), sphingosine species, including sphingosine-1-phosphate and ceramide-1-phosphate, and steroid hormones.

The endoplasmic reticulum plays a central role in sensing and regulating simultaneously lipid homeostasis and protein homeostasis (Volmer and Ron, 2015). The unfolded protein response, the stress-induced quality control system of the endoplasmic reticulum, is activated not only by the accumulation of unfolded or misfolded proteins in the lumen of the endoplasmic reticulum, but also by signaling lipids, including sphingolipids, and by the potentially harmful excess of lipid species such as saturated fatty acids, ceramides and cholesterol (Kono et al., 2017). The capacity to sense aberrant states of both proteins and lipids is mainly carried out by the three master regulators of the unfolded protein response, inositol-requiring enzyme 1 (IRE1α), protein kinase R-like endoplasmic reticulum kinase (PERK), and activating transcription factor 6 (ATF6). These transducers initiate transcriptional responses that balance the relative rates of the biosynthesis and degradation of proteins and lipids (Kono et al., 2017). For example, the generation of lipids is controlled by lipid-composition sensors that regulate the activity of the enzymatic pathways involved in lipid synthesis and degradation to maintain the balance in the lipidome composition and localization in the cell (Puth et al., 2015). In another example, the coordination of the stress response between the mitochondria and cytosol is associated with an increase in fatty acid synthesis and in a decrease in ceramide synthesis in mitochondria, which promotes the expression of the molecular chaperone Hsp70 in the cytosol (Kim et al., 2016).

More generally, molecular chaperones are key players not only in the protein homeostasis system as protein chaperones, but also in the lipid homeostasis system as lipid chaperones. The ability to modulate the physical state of lipid membranes has also been reported not just for small heat shock proteins, Hsp60, Hsp70, and Hsp90 (Balogi et al., 2019). These molecular chaperones are capable of becoming incorporated in lipid membranes through their ability of binding hydrophobic groups, thus stabilizing the conformational properties of lipid membranes upon stress, such as the changes in fluidity caused by temperature fluctuations. For instance, Hsp70 inhibits lysosomal permeabilization induced by diverse stimuli as cytokines and oxidative stress (Nylandsted et al., 2004).

Lipid composition is also maintained by lipid transport through lipoproteins (Van Meer et al., 2008), and lipid storage through lipid droplets (Walther and Farese, 2012; Olzmann and Carvalho, 2019). The regulation of both lipoproteins and lipid droplets requires a cooperation between the lipid and protein homeostasis systems. Lipoproteins, which are secreted particles that transport lipids through plasma circulation, consist of a core of cholesterol esters and triglycerides enclosed in a coat of phospholipids and apolipoproteins. Apolipoproteins are proteins that serve not only as structural components of lipoproteins, but also as ligands for cell-surface receptors and lipid-transport proteins, and cofactors for lipid-processing enzymes. Lipid droplets are structurally similar to lipoproteins as they have micellar structures that consist of a phospholipid monolayer that surrounds a core of neutral lipids, including in particular sterol esters and triacylglycerols (Walther and Farese, 2012). Intracellular membrane trafficking between organelles takes place through a system of diffusing lipid droplets operated by lipid transfer proteins (Wong et al., 2019), or through direct exchange at membrane contact sites between organelles, in which lipid transfer proteins shuttle lipids from one organelle to another (Lahiri et al., 2015). The secretory pathway, including the endoplasmic reticulum, the Golgi and the endosomal system play central roles in this trafficking (Figure 1).

The interplay between the quality control systems of lipids and proteins controls the composition of lipid membranes also at the local level. Lipid rafts are formed by preferential interactions between lipids that generate domains of specific lipid compositions to drive the sorting of membrane proteins. In addition, lipid membranes regulate their fluidity in response to temperature changes through embedded thermosensors (Mendoza, 2014). Membrane tension is kept stable through mechanosensors, which change their conformations depending on membrane bending. For instance, mechanical perturbations of lipid rafts activate the enzyme phospholipase D2 to produce phosphatidic acid, which acts as a signaling lipid (Petersen et al., 2016).

Lipid composition also plays a central role in redox homeostasis, which is the cellular system that controls the balance of oxidative and reducing reactions (Gaschler and Stockwell, 2017). Reactive oxygen species are common redox signaling species, many of which are produced by mitochondria. These species include lipid peroxides, which can diffuse through lipid membranes and serve as lipid signals (Higdon et al., 2012).

The lipid and protein homeostasis systems also control the catabolism of lipids, including through the process of the process of β-oxidation of fatty acids in mitochondria and peroxisomes, which generates acetyl-CoA, a key substrate in the tricarboxylic acid (TCA) cycle. These fatty acids are first generated by lipolysis of triacylglycerol in lipid droplets, by the enzymes adipose triglyceride lipase (ATGL), hormone-sensitive lipase (HSL), and monoacylglycerol lipase (MGL), and then transported to mitochondria and peroxisomes. In turn, eicosanoids can modulate gene expression by binding to lipid-sensing transcription activators, such as the peroxisome proliferator-activated receptors (PPARs), which are nuclear receptors that regulate the expression of genes required for peroxisome activity.

## Dysregulation of Lipid Homeostasis and Its Effects on the Protein Homeostasis System

**FIGURE 2:**
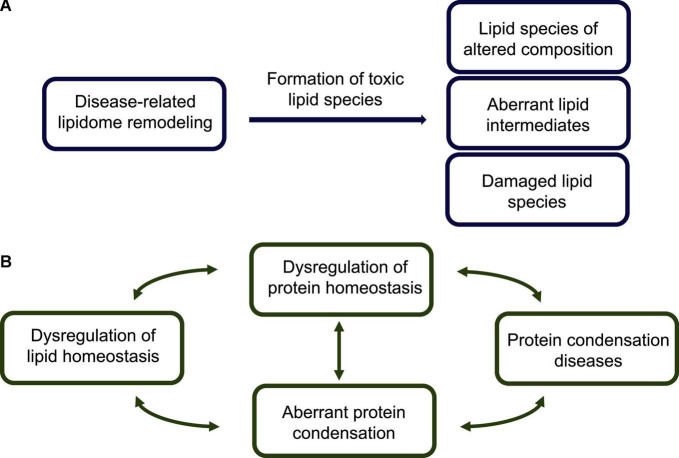
Lipid homeostasis dysregulation, toxic lipid species, and their links to protein misfolding diseases. **(A)** The disease-related impairment of the lipid homeostasis system is associated with dysfunctional interactions of lipids with lipid-processing enzymes (e.g., synthases, transferases and lipases), lipid receptors (e.g., G-protein coupled receptors) and transcription factors (e.g., nuclear receptors), and results in aberrant lipidome remodeling. A modified lipidome exhibits the presence of a variety of potentially toxic lipid species, including: (1) lipid membranes, lipid droplets and lipoproteins of altered compositions, which impair lipid trafficking, (2) accumulated lipid intermediates, including partially metabolized fatty acids, and (3) damaged lipid species, such as peroxidated lipids and advanced lipoxidation end products. **(B)** According to the lipotoxicity hypothesis, possible routes to the development of protein misfolding diseases start from an altered lipidome, which can create cellular conditions conducive to aberrant protein assembly. This altered lipidome can both directly interact with the condensing proteins, and indirectly affect the protein homeostasis system and impair its ability to control the protein condensation process. In essence, the appearance of toxic lipid states can facilitate the formation of toxic protein states, triggering a cascade of pathological events leading to protein misfolding diseases.

Failures in the lipid homeostasis system lead to changes in composition of the lipidome and formation of toxic lipid species (Figure 2A), which in turn can compromise the protein homeostasis system and generate toxic protein states (Figure 2B). At the genome level, these failures may be caused by genetic mutations (Barroso and McCarthy, 2019) or be associated with the molecular damage that accumulates with disease and ageing (Hayflick, 2007). It has been estimated that over 100,000 chemical lesions occur daily within the human genome, which despite the highly efficient DNA repair pathways present in human cells, may results in somatic DNA mutations (Keogh et al., 2018). These damages are particularly relevant in neurons, which being post-mitotic, cannot be readily replaced.

At the lipidome level, a common form of lipotoxicity is caused by elevated levels of circulating fatty acids, which is linked to various diseases (Bazinet and Layé, 2014). Excess fatty acids can be converted abnormally in incompletely metabolized acylglycerols, phospholipids, sphingolipids, and signaling lipids, resulting in the accumulation of intermediate reactive species that induce the release of inflammatory cytokines and reactive oxidative species (Schaffer, 2003). These processes are associated with the metabolic syndrome, a condition that contributes to cardiovascular diseases, obesity, and diabetes, and is estimated to affect perhaps a fourth of the population of Western countries (Unger et al., 2010). In turn, the presence of reactive oxidative species induces the formation, in particular from polyunsaturated fatty acids, of peroxidated lipids. At the proteome level, these species may perturb protein homeostasis, as they contain nucleophilic groups such as aldehydes, ketones and epoxides that can generate advanced lipoxidation end products, by forming adducts with proteins in a process called lipoxidation (Afonso and Spickett, 2019).

A closely related form of lipotoxicity is associated with the dysregulation of lipid droplets (Greenberg et al., 2011; Olzmann and Carvalho, 2019). As excessive levels of free fatty acids can lead to the generation of toxic metabolites and compromise membrane integrity, they are turned into triacylglycerols and incorporated into lipid droplets, whose aberrant accumulation in adipocytes and in hepatocytes is associated with obesity and non-alcoholic fatty liver disease, respectively (Olzmann and Carvalho, 2019). Emerging evidence indicates that defects in lipid processing in lipid droplets is closely linked to damage to the protein homeostasis system. For example, in response to the presence of reactive oxygen and nitrogen species, lipid droplets can release eicosanoids, such as leukotrienes and prostaglandins, which are inflammatory mediators (Marschallinger et al., 2020). More generally, excessive levels of unesterified lipids, such as cholesterol and fatty acids, may exceed the storage capacity of lipid droplets, triggering chronic inflammatory responses, which are often observed in obese people (Walther and Farese, 2012).

## Effects of Lipids on Protein Misfolding, Liquid-Liquid Phase Separation and Amyloid Aggregation

**FIGURE 3:**
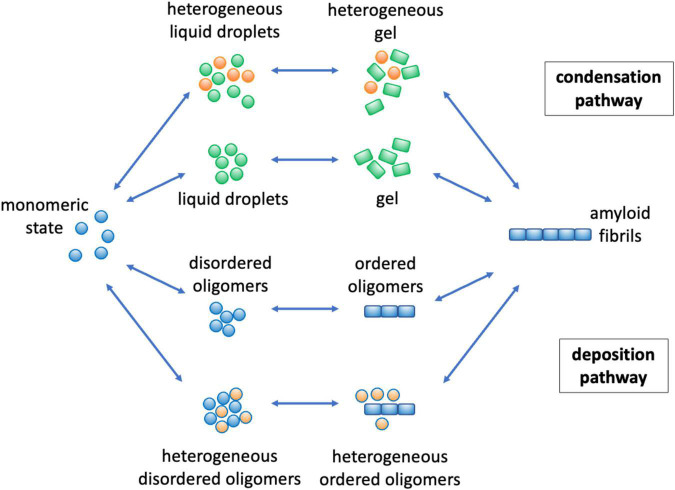
Mechanisms by which lipids can affect protein aggregation through the deposition and condensation pathways. The protein aggregation process can take place through two major pathways. In the deposition pathway (Knowles et al., 2014; Selkoe and Hardy, 2016) (lower part) monomeric proteins initially self-assemble into disordered oligomeric species, while in the condensation pathway (Hyman et al., 2014; Banani et al., 2017; Boeynaems et al., 2018) (upper part) the initial formation of liquid droplets facilitate the formation of disordered oligomers. These early disordered oligomers can then convert into more ordered forms (Cremades et al., 2012; Michaels et al., 2020), which can grow into amyloid fibrils. The overall aggregation process can be facilitated, both thermodynamically and kinetically (Box 1), by the presence of lipids (in orange) both in the deposition (Habchi et al., 2018) and in the condensation (Ray et al., 2020; Hardenberg et al., 2021) pathways. Kinetically, lipids may catalyze the different conversion steps, and thermodynamically they can stabilize heterogeneous species of mixed composition.

Lipids can affect the conversion of proteins into the amyloid state by modulating the protein aggregation network in a variety of ways (Figure 3). In the deposition pathway, the protein aggregation process proceeds through a series of microscopic steps, which begin with the formation of small disordered aggregates through primary nucleation events in which monomeric proteins come together (Knowles et al., 2014). These initial soluble aggregates convert into more ordered species, which can then grow into amyloid fibrils (Knowles et al., 2014). In its homogeneous version, this process happens spontaneously in the absence of co-factors, while in its heterogeneous version, the various steps, including in particular the initial nucleation events, are triggered by cellular factors, which may include lipids, metal ions and metabolites (Figure 3).

More recently, it has been realized the phenomenon of liquid-liquid phase separation can open another route to the formation of amyloid assemblies (Hyman et al., 2014; Banani et al., 2017; Boeynaems et al., 2018; Box 1). In this process, referred to as the condensation pathway in Figure 3, proteins initially phase separate reversibly into a dense liquid phase, which is associated with the formation of membraneless organelles. Under certain conditions, there could be primary nucleation events within the dense liquid phase, which can then give rise to the amyloid pathway. Also this process can be homogeneous or heterogeneous, depending on whether other cellular factors participate in it.

Along both the deposition and condensation pathways, lipids are emerging as potent promoters of protein aggregation (Galvagnion et al., 2015; Habchi et al., 2018; Ray et al., 2020; Hardenberg et al., 2021). In particular, the age-related impairment of lipid homeostasis results in a remodeling of the lipidome, including in particular alterations in the composition of lipid membranes and lipid droplets, and loss of the chemical integrity of the lipid themselves. These changes results in the appearance of aberrant toxic lipid species that can lead to enhanced rates of protein aggregation. In the following we illustrate these points for α-synuclein in Parkinson’s disease and Aβ in Alzheimer’s disease.

## Effects of Lipids on α-Synuclein Aggregation in Parkinson’s Disease

Parkinson’s disease is the most common neurodegenerative movement disorder, with motor symptoms that include tremor, rigidity and bradykinesia (Balestrino and Schapira, 2020). At the pathological level, this disease is characterized by a loss of dopaminergic neurons in the *pars compacta* of the *substantia nigra* and by an accumulation of aberrant inclusions known as Lewy bodies, of which α-synuclein is one of the primary components (Spillantini et al., 1997). The misfolding and aggregation of α-synuclein, which is a peripheral membrane protein highly abundant at synaptic terminals (Spillantini et al., 1997), is thus closely associated with Parkinson’s disease and related synucleinophaties, which include dementia with Lewy bodies and multiple system atrophy (Wong and Krainc, 2017). As one of its physiological functions is to facilitate vesicle trafficking (Murphy et al., 2000; Wong and Krainc, 2017; Fanning et al., 2020b), including by promoting the clustering of synaptic vesicles (Murphy et al., 2000; Fusco et al., 2016; Sun et al., 2019) and the dilation of the exocytotic fusion pore (Logan et al., 2017), α-synuclein is interacting with lipid membranes in a transient and reversible manner. The dysregulation of this type of interaction can trigger its misfolding and aggregation (Galvagnion et al., 2015, 2016). As lipidomic studies have identified changes in the lipidome in Parkinson’s disease brains (Wood et al., 2018), it is important to investigate to extent to which these changes may affect the aggregation of α-synuclein.

**FIGURE 4:**
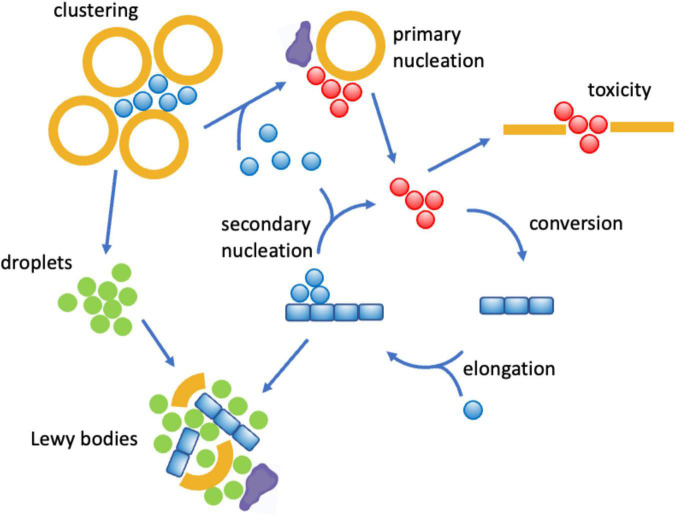
Mechanisms by which lipid dysregulation can affect α-synuclein aggregation in Parkinson’s disease. One of the proposed physiological functions of α-synuclein (blue) is to bind reversibly synaptic vesicles (orange) and to promote their clustering (Murphy et al., 2000; Fusco et al., 2016). However, when multiple α-synuclein molecules bind synaptic vesicles or other lipid assemblies of altered compositions, including exosomes (Danzer et al., 2012; Grey et al., 2015) and lipid droplets (Cole et al., 2002; Soste et al., 2019), they can self-assemble into disordered oligomers (red), which can be cytotoxic by disrupting lipid membranes (Danzer et al., 2007; Fusco et al., 2017), or progress to form amyloid fibrils and Lewy bodies. All these steps can be promoted by lipid membranes of specific compositions (Buell et al., 2014; Galvagnion et al., 2015, 2016) and regulated by interactions with molecular chaperones (purple). The clustering of synaptic vesicles can also open up the condensation pathway by concentrating α-synuclein molecules (Ray et al., 2020; Hardenberg et al., 2021), which may end up in the formation of Lewy bodies through the formation of α-synuclein droplets (Hardenberg et al., 2021) (green).

Many studies have focused on the deposition pathway of α-synuclein aggregation, which involves a network of microscopic processes that include primary nucleation, conversion, elongation and secondary nucleation (Buell et al., 2014; Galvagnion et al., 2015, 2016; Figure 4). While the spontaneous primary nucleation of α-synuclein is a slow process unlikely to play a relevant role in disease, lipids can promote it, as it happens on the surface of lipid membranes (Cremades et al., 2012; Buell et al., 2014; Galvagnion et al., 2015, 2016). Membrane-bound oligomers are produced in this way, and they can then either redissolve or convert into more ordered oligomers and then can grow into amyloid fibrils (Cremades et al., 2012). Oligomers with a high degree of cross-β ordering have been shown to be particularly cytotoxic by being able to penetrate lipid membranes (Danzer et al., 2007; Fusco et al., 2017). It has also been observed that lipid molecules can be incorporated into the aggregated species (Hellstrand et al., 2013), with membrane-bound oligomers being particularly cytotoxic (Killinger et al., 2019). While these events take place intracellularly, aggregated species can exit the cell and spread to neighboring cells (Uemura et al., 2020). Lipids can facilitate also this process, and in particular exosomes have been shown to promote α-synuclein aggregation and spreading (Danzer et al., 2012; Grey et al., 2015). It has also been shown that the inhibition of the interactions between α-synuclein and the molecular chaperones Hsc70 and Hps90 promotes membrane binding and triggers the localization and aggregation of α-synuclein on mitochondria (Burmann et al., 2020).

More recently, it has been reported that α-synuclein can also undergo aggregation through the condensation pathway, which involves a liquid-liquid phase separation step (Ray et al., 2020; Hardenberg et al., 2021; Box 1). The phase separation process can be promoted by the presence of lipids, which remain then incorporated in the condensates, suggesting a possible mechanism of formation of Lewy bodies (Hardenberg et al., 2021), which could explain the complex composition of these deposits (Shahmoradian et al., 2019; Mahul-Mellier et al., 2020; Trinkaus et al., 2020).

Overall, a close relationship is emerging between the impairment of lipid homeostasis and the onset and progression of Parkinson’s disease (Killinger et al., 2019; Fanning et al., 2020b; Tripathi et al., 2022). A variety of genetic mutations linked to the disease have revealed proteins that affect lipid vesicle trafficking and composition (Abeliovich and Gitler, 2016; Nalls et al., 2019; Fanning et al., 2020b). The *LRRK2* gene, which is associated with both familial and idiopathic forms of the disease, encodes leucine-rich repeat kinase 2 (LRRK2), a kinase that regulates secretory and endocytic vesicle trafficking by phosphorylating a group of Rab peripheral membrane proteins (Abeliovich and Gitler, 2016; Fanning et al., 2020b). Although further investigations are needed, aberrant vesicle populations and cellular locations may promote the formation and spreading of α-synuclein aggregates, thus linking LRRK2 with lipid homeostasis (Fanning et al., 2020b). Mutations in *GBA*, the gene encoding the enzyme glucocerebrosidase, which plays an important role in the autophagy-lysosome pathway, significantly increase the risk of Parkinson’s disease (Mazzulli et al., 2011; Abeliovich and Gitler, 2016). Glucocerebrosidase catalyzes a crucial step in the lysosomal breakdown of glycosphingolipids into their glucose and ceramide components, allowing them to be recycled to the plasma membrane, where they modulate its properties (Mazzulli et al., 2011; Abeliovich and Gitler, 2016). A decrease in glucocerebrosidase activity induces a decrease in autophagy and promotes α-synuclein aggregation, as well as its release from neurons and transmission between neurons (Gegg et al., 2020; Henderson et al., 2020). In particular, it has been reported that glucosylceramide, one of the substrates of glucocerebrosidase, promotes the toxic formation in human midbrain dopaminergic neurons of α-synuclein oligomers (Zunke et al., 2018), consistent with the observations that the composition of lipid membranes controls the initial events in α-synuclein aggregation (Buell et al., 2014; Galvagnion et al., 2015, 2016). The complexity of the interplay between protein and lipid homeostasis is also illustrated by the emerging relationship between lysosomal storage disorders and Parkinson’s disease, at least in part through the consequences of reduced glucocerebrosidase activity in lysosomes (Mazzulli et al., 2011), where one may expect the low pH to promote the proliferation of α-synuclein aggregates by secondary nucleation (Buell et al., 2014), which could follow the glycosphingolipid-dependent formation of initial α-synuclein seeds (Mazzulli et al., 2011; Zunke et al., 2018).

More generally, the chemical properties of lipids, including the types and charges of the head groups, and the length and saturation state of the hydrocarbon chains, which control membrane fluidity, have been identified as a determinant of membrane-induced α-synuclein aggregation (Galvagnion et al., 2016). In addition, membrane packaging defects, which are promoted by high curvature and fluidity, favor α-synuclein binding (Nuscher et al., 2004). These observation help understand the identification through genome-wide association studies (GWAS) (Nalls et al., 2019) of enzymes in the lipid homeostasis system that modulate the physico-chemical properties of lipids, including the fatty acid elongase ELOVL7, which controls the length of the hydrocarbon chains, and the phospholipase PLA2G6, which modifies phospholipid head groups and free fatty acids by hydrolyzing acyl and phosphate esters (Fanning et al., 2020b).

Furthermore, lipid droplets may provide a scaffold for α-synuclein aggregation and for the formation of Lewy bodies (Cole et al., 2002; Soste et al., 2019). This observation is linked with the suggestion that α-synuclein may play a part in the lipid homeostasis system by acting as a perilipin, participating in the formation of lipid droplets (Soste et al., 2019). Thus, the dysregulation of lipid droplet homeostasis may enhance α-synuclein aggregation. In this context, it has been observed in yeast that the upregulation of diacylglycerol results in increased formation of lipid droplets and, correspondingly, increased α-synuclein aggregation (Soste et al., 2019). In turn, the pharmacological inhibition of phosphatidic acid phosphohydrolase 1, a phosphatase involved in the synthesis of diacylglycerol, reduced α-synuclein-induced toxicity (Soste et al., 2019).

It has also been observed in mesencephalic neuronal cells that increasing levels of polyunsaturated fatty acids can promote the formation of α-synuclein oligomers while the opposite is seen for saturated fatty (Sharon et al., 2003), providing support to the idea that alterations of lipid homeostasis can affect α-synuclein aggregation. Conversely, α-synuclein can modulate lipid homeostasis, as shown by a recent lipidomic study in which the overexpression of this protein in yeast led to an increase in the production of diglycerides, triglycerides and unsaturated fatty acids, and in turn in the accumulation of lipid droplets (Fanning et al., 2019). This effect could be mitigated by the inhibition of stearoyl-CoA desaturase, an enzyme that catalyzes the formation of monounsaturated fatty acids from saturated fatty acids (Fanning et al., 2019). Decreasing monounsaturated fatty acids in this way was seen to reduce α-synuclein aggregation, protect dopaminergic neurons, and prevent progressive motor deficits in a mouse model of Parkinson’s disease (Nuber et al., 2021). These results indicate that targeting fatty acid desaturases may represent a promising route for therapeutic intervention for this disease (Nuber et al., 2021).

## Effects of Lipids on Aβ Aggregation in Alzheimer’s Disease

Alzheimer’s disease is the most common cause of dementia, a condition that affects over 50 million people worldwide (Patterson, 2018). At the molecular level, this disease is characterized by the presence of amyloid plaques, which are aberrant deposits formed primarily by the Aβ peptide in the brains of affected individuals (Selkoe and Hardy, 2016; Jack et al., 2018). According to the amyloid hypothesis, the formation of these deposits triggers a series of pathological events that ultimately result in synaptic loss and neuronal death (Balch et al., 2008; Knowles et al., 2014; Selkoe and Hardy, 2016; Hampel et al., 2021a). In a small fraction of cases, the aggregation of Aβ is enhanced by familial mutations, causing the early onset of the disease (Selkoe and Hardy, 2016). More commonly, however, the disease is associated with an age-related impairment of Aβ homeostasis, which involves perturbations in Aβ synthesis, trafficking and degradation (Balch et al., 2008; Knowles et al., 2014; Selkoe and Hardy, 2016). The deterioration of Aβ homeostasis can also result from an altered cellular environment that becomes more conducive to Aβ aggregation in ways that we are beginning to understand. In particular, lipidomic studies have revealed widespread disease-related changes in the human lipidome in Alzheimer’s disease, in particular in phospholipids, cholesterol, and triglycerides (Di Paolo and Kim, 2011), and identified specific lipids as biomarkers of the disease, including in particular a set of phosphatidylcholine metabolites (Mapstone et al., 2014).

**FIGURE 5:**
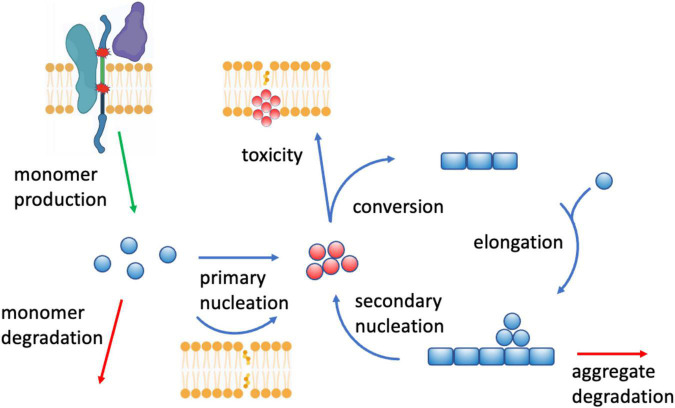
Crosstalk between perturbed lipid homeostasis and Aβ aggregation in Alzheimer’s disease. There are at least three aspects of this crosstalk: (1) lipids affect Aβ homeostasis in a number of ways, for example by modulating the concentration Aβ monomers by regulating the activity of the secretases (top left) involved in the proteolytic cleavage of Aβ from APP (Di Paolo and Kim, 2011; Haass et al., 2012; Crivelli et al., 2020) (“monomer production”), of the endopeptidases (“monomer degradation”) that degrade Aβ monomers (Yao et al., 2008), and of the endolysosomal system that removes Aβ aggregates (Morel et al., 2013) (“aggregate degradation”), (2) lipid membranes of altered composition (cholesterol (Habchi et al., 2018) and gangliosides (Matsuzaki, 2020)) can trigger Aβ aggregation by promoting the formation of disordered oligomers (in red) “primary nucleation”), which then grow into amyloid fibrils (Michaels et al., 2020) (“conversion,” “elongation,” and “secondary nucleation”), and (3) Aβ aggregates, including oligomers, can in turn compromise lipid homeostasis, including by perturbing lipid membranes by binding to them (“toxicity”), or by disrupting lipid signaling by binding membrane receptors (Haass and Selkoe, 2007).

Increasing evidence that indicates that lipids can be triggers, modulators and targets of Aβ aggregation. To understand the role of lipids as triggers of Aβ aggregation, one could start from the observation that this process involves a network of microscopic steps, including the primary nucleation of monomers into misfolded oligomers, the conversion of these oligomers into more ordered growth-competent species, the elongation of fibrils, and the secondary nucleation of monomers in a fibril-catalyzed manner (Knowles et al., 2014; Michaels et al., 2020; Figure 5). Lipids, in particular cholesterol (Habchi et al., 2018) and gangliosides (Matsuzaki, 2020), can promote the primary nucleation of Aβ, an event that at the normal Aβ brain concentrations is otherwise likely to be rare (Habchi et al., 2018). More broadly, different lipid species can have very different effects on aggregation, ranging from inhibition to promotion (Ryan et al., 2011). The presence of these opposite effects has prompted the investigation of the mechanisms that prevent lipid membranes from triggering Aβ aggregation. It has been suggested that the complexity in the composition of lipid membranes represent a protective mechanism against Aβ aggregation by buffering extreme behaviors. In this ‘resilience by complexity’ mechanism, the effect of aggregation-promoting phospholipids, such as phosphatidylcholine and phosphatidylethanolamine, is balanced by the aggregation-inhibiting effects of other phospholipids, including phosphatidylglycerol and phosphatidylserine (Sanguanini et al., 2020). These findings suggest the intriguing possibility that different cellular membranes, depending on their lipid composition and its dysregulation upon the age-related remodeling of the lipidome, may exhibit different vulnerability to Aβ aggregation and play different roles in the onset of Alzheimer’s disease.

Lipids can also act as modulators of Aβ production and aggregation. Aβ is generated by proteolytic cleavage from the amyloid precursor protein (APP), a process carried by the β- and γ-secretases (Selkoe and Hardy, 2016). This process may occur along the secretory pathway of APP, including in the early endosomes and lysosomes (Haass et al., 2012). As secretases are membrane-bound enzymes, their activity can be regulated by the composition of the lipid membranes, and indeed a variety of lipids and of regulators of lipid homeostasis have been shown to influence this process (Di Paolo and Kim, 2011). For example, the non-amyloid proteolytic processing of APP by α-secretase, which generates the less pathogenic p3 peptide rather than Aβ (Haass et al., 2012), is promoted by isoprenoids, diacylglycerol and phospholipase C, while cholesterol, ceramide and sphingosine-1-phosphate promote the activity of β-secretase (Crivelli et al., 2020). In addition, cholesterol and sphingolipids promote γ-secretase activity, while docosahexaenoic acid, sphingomyelinase and phospholipase D1 inhibit it (Di Paolo and Kim, 2011). Thus, APP processing occurring within the cholesterol-rich lipid rafts appears to be potentially amyloidogenic (Di Paolo and Kim, 2011). Furthermore, cholesteryl esters, the storage product of excess cholesterol, bind the cholesterol-binding domain of APP and regulate Aβ secretion (van der Kant et al., 2019). These observations indicate how the composition of lipid membranes can affect the activity of membrane enzymes through the modulation of properties of their environment.

Furthermore, lipidomics, transcriptomics, and proteomics studies converge on the impairment of protein degradation pathways in Alzheimer’s disease, including the ubiquitin-proteasome and the endolysosomal systems (Di Paolo and Kim, 2011), resulting in the progressive accumulation of supersaturated proteins at risk of aggregation (Kundra et al., 2017). Since lipids play integral structural and signaling roles in these degradation systems, they can influence them, as for example illustrated by the finding that the endosomal trafficking is controlled by phosphatidylinositol-3-phosphate, which is selectively deficient in brain tissue in Alzheimer’s disease patients (Morel et al., 2013). The degradation of Aβ can be influenced by lipids also in other ways, as shown for example by the finding that neprilysin, one of the most important Aβ endopeptidase, is upregulated by steroid hormones, resulting in decreased Aβ levels (Yao et al., 2008).

In turn, lipids can be perturbed by Aβ aggregation. The relationship between lipid membranes and Aβ aggregation is bidirectional, as the aggregation process of Aβ produces highly cytotoxic soluble intermediates, including oligomers and protofibrils. These species can disrupt lipid membranes making them permeable to calcium ions, and interact in aberrant manners with membrane receptors, triggering in particular inflammatory responses (Haass and Selkoe, 2007; Figure 5). Aβ can further perturb lipid homeostasis by its ability to bind metal ions, which can catalyze the generation of lipid peroxidation products using polyunsaturated fatty acids as electron donors (Di Paolo and Kim, 2011).

In connection to these findings, GWAS have identified several genes encoding proteins involved in lipid homeostasis (Lambert et al., 2013). These studies have strongly implicated the ε4 allele of the *APOE* gene, which encodes the E4 isoform of apolipoprotein E, as a major risk factor for Alzheimer’s disease. Although the association of this isoform with disease is complex and incompletely understood, it appears to be slightly defective in regulating triglyceride, phospholipid and cholesterol transport (Yamazaki et al., 2019), thus conceivably resulting in the formation of lipid membranes of more aggregation-promoting compositions. Consistent with this mechanism of action, reduced levels of ATP-binding cassette (ABC) transporter A7 (ABCA7), which is encoded by the GWAS *ABCA7* gene and mediates the efflux of cholesterol and phospholipids to form high-density lipoproteins, correspond to increased lipotoxicity (Lyssenko and Praticò, 2021) and Aβ aggregation (De Roeck et al., 2019). *SORL1*, another GWAS gene, encodes an apolipoprotein E receptor, which has also emerged from a combined lipidomic and proteomic study of Alzheimer’s disease (Xu et al., 2020). Three other GWAS genes involved in lipid metabolism are *CLU, TREM2* and *PLCG2*. *CLU* encodes clusterin, a molecular chaperone involved in Aβ clearance and in cholesterol metabolism and trafficking, and *TREM2*, which encodes a receptor primarily expressed in microglia that can be activated by damage-associated lipids and trigger the removal of Aβ plaques (Wang et al., 2015). By using cell-specific proteomics, it has been recently reported that *TREM2* deficiency leads to a disruption of microglial lipid metabolism with cholesteryl ester accumulation (Lewcock et al., 2020; Nugent et al., 2020). A related role has been described for *PLCG2*, which encodes phospholipase Cγ2, a lipid-processing enzyme that cleaves phosphatidylinositol-4,5-bisphosphate to diacylglycerol and inositol-1,4,5-trisphosphate (Andreone et al., 2020; Takalo et al., 2020).

## Challenges and Opportunities Ahead

Although the lipid and protein homeostasis systems have been intensely studied, they have been so far considered in a relatively disconnected manner. A greater recognition of their interdependence will stimulate investigations of the mechanisms by which these systems cooperate under normal conditions and cause pathological feedback processes in disease states (Xu et al., 2020; Hampel et al., 2021b). This knowledge will facilitate the characterization of the interplay between the different branches of the cellular homeostasis system. It is becoming clear that the overall balance of the cellular processes depends on the cooperation of the systems that regulate the cellular components, including not only lipids and proteins, but also metabolites, carbohydrates and nucleic acids. Although the corresponding homeostasis systems have distinctive features, they interact and overlap to a great extent.

It will be particularly important to identify in more detail toxic states of lipids and proteins, and to characterize the mechanisms by which they exert their pathological effects. Although we have acquired knowledge about some toxic lipid states, including lipid membranes and lipid droplets of aberrant compositions, and peroxidated lipids (Figure 2), as well as about some toxic protein states, including misfolded oligomers (Figures 3–5), it is likely that additional toxic states will emerge, providing novel insights and mechanistic details of the connections between proteotoxicity and lipotoxicity.

In parallel, the increasing recognition of the widespread presence of phase separation phenomena in cell biology is also revealing a broad range of previously unsuspected processes, in which liquid-like states dense in specific components play important roles in both physiological and pathological mechanisms (Hyman et al., 2014; Banani et al., 2017; Boeynaems et al., 2018; Fuxreiter and Vendruscolo, 2021; Box 1).

Advancing our knowledge of all of these processes will require a better understanding of the causal relationships within a network. The organization of the cellular homeostasis system is not linear, but has a web structure. In this type of system, causal relationships are complex, as feedback mechanisms may turn effects into causes, as for example in the case of the Aβ aggregation process, where amyloid fibrils are not only the end product of the process itself, but also act as catalysts for the formation of new aggregates (Michaels et al., 2020; Figure 5).

An increase in our knowledge of the structure of the cellular homeostasis network will suggest novel routes to diagnostic and therapeutic interventions to treat diseases associated with the impairment of the lipid and protein homeostasis systems. The analysis of the structures of biological networks has revealed a hierarchical structure in which certain components act as hubs, being highly connected, while others are specific of sub-networks (Barabási et al., 2011). To achieve specificity, both in diagnostics and therapeutics, it is likely that convenient targets could be chosen among these latter components, in order to avoid perturbing multiple processes.

Looking ahead, with the advent of lipidomics and proteomics, as well as of genomics, transcriptomics and metabolomics, we are acquiring unprecedented knowledge about the cellular components and of their relationships. By building on this knowledge, in the coming years we can expect great advances in our ability to identify the cellular processes whose dysregulation causes diseases associated with aberrant protein misfolding, and to develop therapeutic interventions to treat these conditions.

## Author Contributions

MV wrote the review manuscript.

## Conflict of Interest

The author declares that the research was conducted in the absence of any commercial or financial relationships that could be construed as a potential conflict of interest.

## Publisher’s Note

All claims expressed in this article are solely those of the authors and do not necessarily represent those of their affiliated organizations, or those of the publisher, the editors and the reviewers. Any product that may be evaluated in this article, or claim that may be made by its manufacturer, is not guaranteed or endorsed by the publisher.
